# Analysis of the Dominant Effects Mediated by Wild Type or R120G Mutant of αB-crystallin (HspB5) towards Hsp27 (HspB1)

**DOI:** 10.1371/journal.pone.0070545

**Published:** 2013-08-12

**Authors:** Stéphanie Simon, Valeriya Dimitrova, Benjamin Gibert, Sophie Virot, Nicole Mounier, Mathieu Nivon, Carole Kretz-Remy, Véronique Corset, Patrick Mehlen, André-Patrick Arrigo

**Affiliations:** 1 Hôpital Henri Mondor University, Créteil, France; 2 Department of Clinical Research, Division of Pediatric Hematology/Oncology, Insel Spital, Institute of Pathology, Bern University, Bern, Switzerland; 3 CGphiMC, CNRS UMR 5534, Claude Bernard University Lyon 1, Villeurbanne, France; 4 Apoptosis Cancer and Development Laboratory, Lyon Cancer Research Center, INSERM U1052-CNRS UMR5286, Centre Léon Bérard, Claude Bernard University Lyon 1, Lyon, France; University of Durham, United Kingdom

## Abstract

Several human small heat shock proteins (sHsps) are phosphorylated oligomeric chaperones that enhance stress resistance. They are characterized by their ability to interact and form polydispersed hetero-oligomeric complexes. We have analyzed the cellular consequences of the stable expression of either wild type HspB5 or its cataracts and myopathies inducing R120G mutant in growing and oxidative stress treated HeLa cells that originally express only HspB1. Here, we describe that wild type and mutant HspB5 induce drastic and opposite effects on cell morphology and oxidative stress resistance. The cellular distribution and phosphorylation of these polypeptides as well as the oligomerization profile of the resulting hetero-oligomeric complexes formed by HspB1 with the two types of exogenous polypeptides revealed the dominant effects induced by HspB5 polypeptides towards HspB1. The R120G mutation enhanced the native size and salt resistance of HspB1-HspB5 complex. However, in oxidative conditions the interaction between HspB1 and mutant HspB5 was drastically modified resulting in the aggregation of both partners. The mutation also induced the redistribution of HspB1 phosphorylated at serine 15, originally observed at the level of the small oligomers that do not interact with wild type HspB5, to the large oligomeric complex formed with mutant HspB5. This phosphorylation stabilized the interaction of HspB1 with mutant HspB5. A dominant negative effect towards HspB1 appears therefore as an important event in the cellular sensitivity to oxidative stress mediated by mutated HspB5 expression. These observations provide novel data that describe how a mutated sHsp can alter the protective activity of another member of this family of chaperones.

## Introduction

The human small Heat shock proteins family contains 10 members that are characterized by a common alpha-crystallin domain [1]. Four members (HspB1, HspB4, HspB5 and HspB8) have ATP-independent chaperone activity but only three of them (HspB1, HspB5 and HspB8) display enhanced level of expression in response to heat shock or stimuli that misfold and damage polypeptides. sHsps are constitutively expressed in many different tissues. In that regard, HspB1 (also denoted Hsp27) is expressed in most tissues. HspB5 (also denoted αB-crystallin) is also expressed in a wide range of tissues, including lens, heart, skeletal muscle, colon, lung and kidney [2] whereas HspB4 (αA-crystallin) is mainly expressed in the lens [3]. These proteins have the ability to interact with each other and form hetero-oligomeric complexes. For example, in mammalian lenses, HspB4 and HspB5 form a major structural protein complex, denoted α-crystallin, involved in the refractive and light focusing properties of the lens [4]–[7]. This complex is present in both the water soluble and insoluble fractions of the lens, especially in the nucleus region, a domain of the lens where the only other detectable sHsp, particularly in caracteous lens, is the phosphorylated form of HspB1 [8]. In addition, these Hsps share the ability to enhance the resistance of cells to the deleterious effects induced by stresses, such as those induced by heat shock, drugs [9], [10], UV light [11] and alterations in intracellular redox homeostasis [12]–[18]. In that respect, they prevent aggregation and precipitation of misfolded or oxidized proteins [19], [20]. In addition, HspB1 and HspB5 can act as anti-oxidant proteins leading to the establishment of a pro-reducing state in cells [13], [21], [22] by up-regulating the activity of anti-oxidant enzymes, such as glucose 6-phosphate dehydrogenase (G6PDH) [14], [23]. These two chaperones also play anti-apoptotic and tumorigenic roles by interacting with specific key protein partners and are nowadays considered as potent anti-cancer therapeutic targets [24], [25], [26]–[29]. Another major role of HspB1, HspB4 and HspB5 relates to their ability to modulate and stabilize cytoskeleton architecture [30]–[36]. For instance, HspB5 chaperone activity is required to stabilize and modulate intermediate filaments assembly and avoid their aggregation [33]. In that respect, several mutations in HspB5 have been shown to alter cytoskeletal architecture, such as the natural missense mutation R120G, which is responsible for cataracts, cardiomyopathies and desmin-related myopathies [37], [38]. The removal of the positive charge from arginine 120 is known to cause HspB5 partial unfolding, increased exposure of hydrophobic regions, abnormal assemblies and subunit exchange and enhanced susceptibility to proteolysis [39], [40]. The mutation also reduces HspB5 solubility and promotes its aggregation [39], [41]. In addition, it strongly impairs HspB5 chaperone activity [42].

A fundamental property of sHsps is their ability to oligomerize. For example, HspB1 forms dynamic polydispersed structures with heterogenous native sizes comprised between 50 and 800 kDa [43], [44] while HspB5 native size is more uniformly distributed within the 700 to 800 kDa range. The oligomerization of HspB1 is a dynamic phenomenon linked to cell physiology that probably allows HspB1 interaction with specific client proteins [27]. On the other hand, HspB1 large oligomers can also act as reservoirs that store stress-induced misfolded or oxidized polypeptides until they are either refolded by ATP-dependent chaperones (Hsp70 and co-chaperones) or degraded [45], [46]. Another parameter to take into account is the phosphorylation of sHsps. This is a complex phenomenon because these proteins have several serine sites that can be phosphorylated differently depending on cell physiology [47]–[49]. HspB1 is phosphorylated in the N-terminal part of the protein, and therefore outside of the alpha-crystallin domain, at serine sites 15, 78 and 82 by mitogen-activated protein kinases associated protein kinases 2,3 (MAPKAPK2,3) [50]. Similarly, HspB5 is phosphorylated at serines 19, 45 and 59. MAPKAPK2,3 phosphorylates serine 59 whereas serine 45 appears to be controlled by p42/p44 MAPKinase. The kinase responsive of serine 19 phosphorylation of HspB5 is still unknown. Phosphorylation is thought to act as a signaling mechanism regulating sHsps oligomerization [51], [52] since phosphomimetic (Ser to Asp) mutants abolish, at least in cultured cells, HspB1 and HspB5 ability to oligomerize [48], [53]. This assumption is also supported by the fact that HspB1 amino terminus, which is involved in phosphorylation sensitive interactions, is crucial for oligomerization [54]–[56]. Consequently, HspB1 and HspB5 holdase chaperone activity are regulated by the complex relationship that exists between their phosphorylation and oligomerization status [15], [57]. For example, it is particularly intriguing to note that in cells exposed to different environmental conditions or insults, HspB1 displays stress-specific changes in its oligomerization/phosphorylation status [58]. Consequently, HspB1 probably acts as a protein sensor, which through structural changes, can interact with the most appropriate client protein targets [27]. These phenomena subsequently allow cells to adapt to changes in their environment and/or mount a protective anti-stress response.

In tissues that express several sHsps, such as in lens and muscles, these proteins can interact and form multiple combinatorial oligomeric structures that can bear different functions. One example is the 3 to 1 unique large chimeric oligomer formed by HspB4 and HspB5 in lens fiber cells [4]–[6], [59]. This oligomeric structure appears to have a higher stability and to be a more efficient chaperone than the individual polypeptides [59], [60]. Indeed, in spite of their high degree of homology, HspB4 and HspB5 polypeptides are characterized by their conformational and functional differences [61]. HspB5 is, for example, more susceptible than HspB4 to heat-induced conformational change and aggregation [62]. In tissues where HspB1 is expressed along with HspB5, it interacts with HspB5 [63] and may serve, as HspB4 does in the lens, to chaperone and stabilize HspB5 conformation, particularly in stress conditions [64]. Moreover, the subunit exchange between HspB5 and HspB1 is more rapid than between HspB5 and HspB4 [59]. The protective effect of HspB1 is particularly intense towards the R120G mutant of HspB5, an unstable polypeptide prone to aggregate [65]. Of interest, HspB1 increases its chaperone-like activity by interacting with HspB5 [59].

The goal of this study was to examine stable HeLa cell clones that express similar levels of either wild type or R120G mutated HspB5 and endogenous HspB1. We show pronounced and opposite effects induced by wild type and mutant HspB5 on cell morphology and oxidoresistance. The R120G mutation increased the native size of HspB1-HspB5 complex and its resistance to salt-induced dissociation. It also allowed the phosphorylation of HspB1 serine15 in the complex, a modification that stabilized HspB1 interaction with mutant HspB5. In oxidative conditions, the partial dissociation of HspB1-HspB5 complex was drastically enhanced in cells expressing mutant HspB5, a phenomenon followed by the aggregation of the two protein partners. In addition to the chaperone effect of HspB1 towards mutant HspB5, these observations enlighten the major dominant positive (wild type) and negative (mutant) effects of HspB5 towards HspB1 in normal and oxidative conditions.

## Results

### Characterization of HeLa cells expressing wild type or R120G mutant HspB5

**Figure 1:**
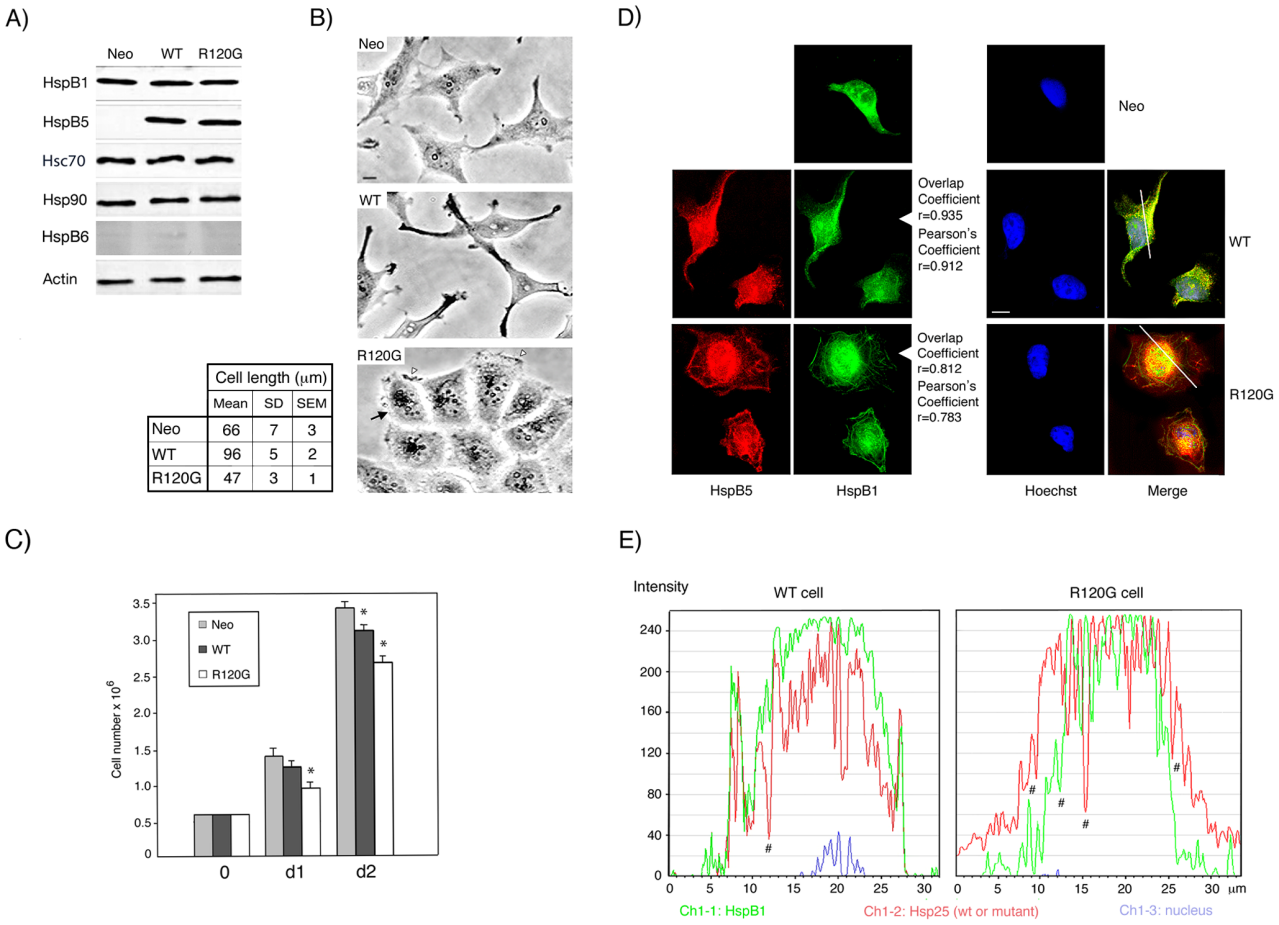
Characterization of Neo, WT and R120G cells. A) Immunoblot analysis of total cellular extracts of Neo, WT and R120G HeLa cells. The levels of HspB1, HspB5, Hsp70, Hsp90, HspB6 and Actin were detected in immunoblots probed with the corresponding antibodies (see Materials and Methods). B) Phase contrast analysis of Neo, WT and R120G. bar: 10 µm. Black arrows: perinuclear granules; white arrowheads: membranous ruffles. Analysis of the biggest dimension of cells (cell length) is presented in the adjacent figure. Mean, SD (standard deviation) and SEM (standard error of mean) of twenty different measurements are presented. C) Analysis of the number of cells in the cultures was from day 0 to days 1 and 2 (d1, d2). Values are means ± SEM of three independent experiments. One-way ANOVA within a time point analysis indicates statistically significant growth differences between Neo and WT and R120G cell lines, *P<0.05. D) Immunofluorescence analysis. Neo, WT and R120G cells were processed for the immunofluorescence detection of HspB5, HspB1 and nuclei as described in Materials and Methods. Bar: 10 µm. Cells were stained for HspB5 (red fluorescence), HspB1 (green fluorescence), nuclei (blue fluorescence) and processed as described in Materials and Methods. The fusion images (Merge) of WT and R20G cells are shown. Overlap and Pearson's coefficients are indicated. E) The graphs represent the fluorescence distribution of HspB1 (green; Ch1-1), wild type or mutant HspB5 (red; Ch1-2) and nucleus (blue; Ch1-3) of the section of WT or R120G cells shown in the green/red fusion images (Merge). #: areas where the co-localization of HspB1 and HspB5 (wild type or mutant) may not occur.

Following transfection and selection, G418 resistant HeLa clones that express either wild type or R120G mutant HspB5 (see Materials and Methods) were analyzed in immunoblots (Fig. 1A). Comparison with the signals generated by serial dilutions of pure HspB5 revealed that two clones (denoted WT and R120G) expressed similar levels (6 ng/µg of total cellular proteins) of wild type or mutant HspB5. Similar analysis, using serial dilutions of pure HspB1, revealed that WT and R120G cells expressed 5 ng of HspB1 per µg of total cellular proteins, a value, also detected in parental and control cells stably transfected with empty vector (Neo cells), that was close to that of HspB5. Moreover, the constitutive expression of wild type or mutant HspB5 did not modify the level of Hsp90, Hsp70, and HspB1 (Fig. 1A), hence suggesting that the presence of exogenous HspB5 polypeptides inside HeLa cells was not sensed as a stress. We also tested for the presence of HspB6, a member of the family of small Hsps that can form chimeric hetero-oligomers with HspB1 in vitro [70], [71]. This protein was barely detectable in Neo, WT and R120G cells (Fig. 1A) and was therefore not further studied. HspB1 and HspB5 appear therefore as the major interacting small Hsps that are present in Neo, WT and R120G cells. We next analyzed of the effects mediated by their interaction inside cells.

Phase contrast analysis of cell morphology revealed that HeLa cells stably expressing wild type HspB5 displayed a drastically elongated appearance (up to 96+/−5 µm) leading to the formation of long, dense and narrow edges (Fig. 1B) that correlated with a polarization of F-actin in the form of stress fibers oriented along the long axis of the cells (See Data S1). This elongated morphology was not observed in cells expressing the R120G mutant of HspB5 (R120G cells are 47+/−3 µm long). These cells had a more compact rounded morphology and a smaller size than Neo or parental HeLa cells (66+/−7 µm). Moreover, they differed in their appearance from WT and control Neo cells by displaying intense perinuclear granulation and dense membranous ruffles (Fig. 1B). They also displayed thick F-actin filaments at the cell periphery where they formed a dense spherical network and collapsed intermediate filaments (See Data S1). These morphological changes correlated with slightly decreased cell number (Fig. 1C), suggesting an effect on cell proliferation or death. Immunofluorescence analysis presented in Fig. 1D, revealed that, in spite of their perinuclear localization, HspB1 and HspB5 also accumulated along the narrow opposite edges that characterize the long axis of WT cells. They also decorated spherical networks at the periphery of R120G cells where F-actin filaments is present. A significant co-localization of HspB1 with HspB5 was detected in merge image that was confirmed by Overlap and Pearson's coefficients (r = 0.9) and computerized image analysis (Fig. 1D,E). However, in R120G cells, the co-localization was not as intense as that observed in WT cells (r = 0.8); a phenomenon that could result of the presence of aggregate-like structures stained by either mutant HspB5 or HspB1. However, these aggregate-like structures were of small size and not comparable, in number and size, to those observed in transiently transfected HeLa cells expressing high loads of mutant HspB5 (See Data S2).

**Figure 2:**
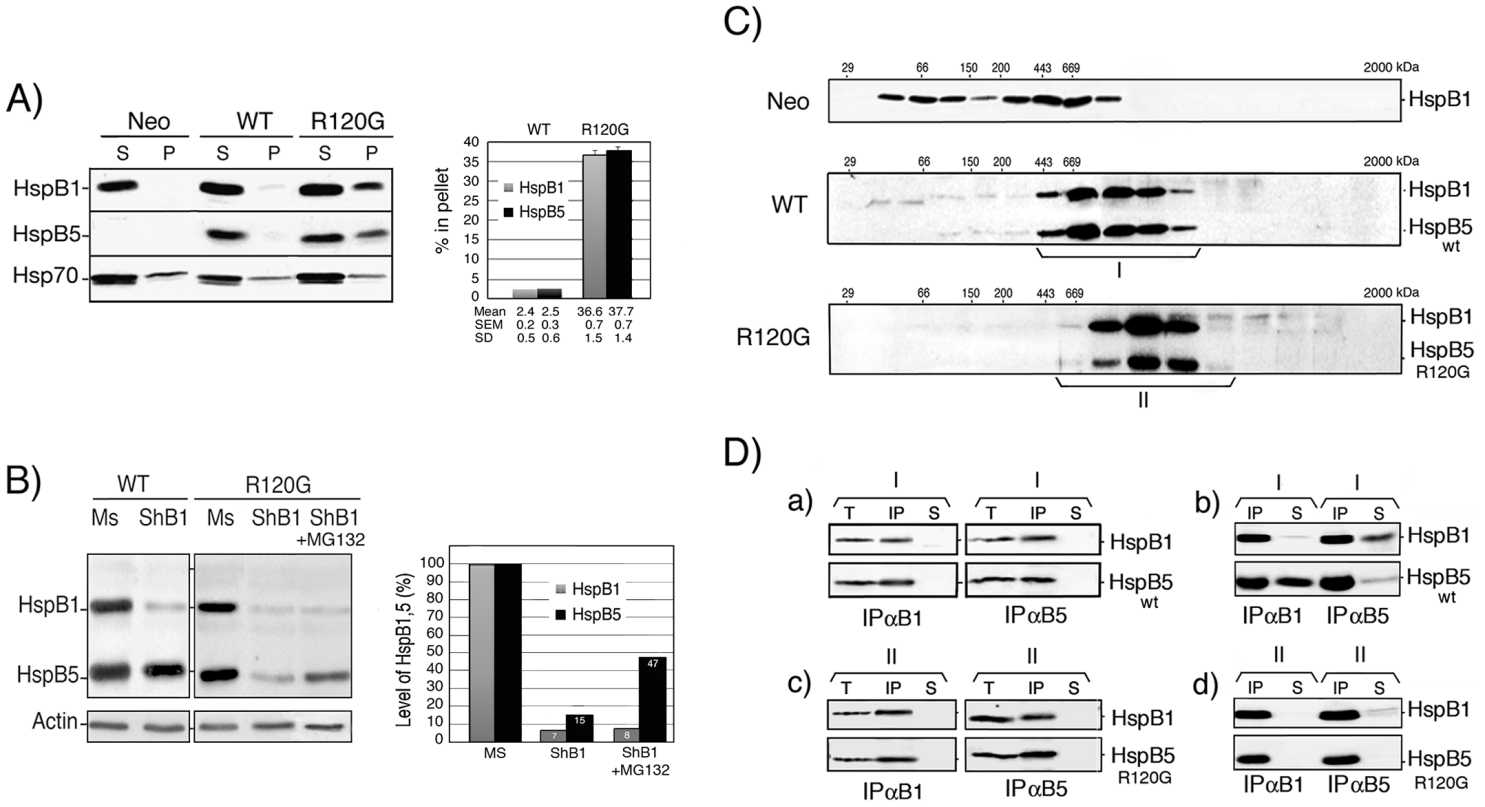
Characterization of HspB1, HspB5 and mutant HspB5 in HeLa cell clones. A) Cellular distribution of HspB1, HspB5 (wild type and mutant) and Hsp70 upon cell lysis. Neo, WT and R120G cells were lysed in the presence of 0.1% Triton X-100 and spun at 10,000×*g* as described in Materials and Methods. The levels of HspB1, HspB5 and Hsp70 present in the supernatant and pellet fractions were detected in immunoblots probed with the corresponding antibodies (see Materials and Methods). Autoradiographs of ECL-revealed immunoblots are presented. Quantitative analysis of three independent experiments is presented in the adjacent figure. B) Effect of shRNA-mediated depletion of HspB1. WT and R120G cells were transiently transfected with control mismatch pSuperNeo-MsRNA27 (Mismatch: Ms) or pSuperNeo-ShRNA27 (ShB1) vector targeting HspB1 mRNA (see Materials and Methods). Two days after transfection, cells were analyzed in immunoblots probed with HspB1, HspB5 and actin antibodies. ShB1 transfected cells were also treated for the last 20 h before being analyzed with 0.5 µmol/l of MG132. Quantitative analysis of one particular experiment where the RNAi-mediated transient depletion of HspB1 was almost complete is presented in the adjacent figure. C) Analysis of HspB1 and HspB5 native sizes in Neo, WT and R120G cells. Cells were lysed as above and the 10,000×*g* cytosolic supernatant fractions were applied to Sepharose 6B gel filtration columns (see Materials and Methods). The presence of HspB1 and HspB5 in pooled fractions eluted from the columns was detected in immunoblots probed with the corresponding antibodies. Autoradiographs of ECL-revealed immunoblots are presented. 29, 66, 150, 200, 443, 669 kDa are gel filtration markers. Exclusion size of the column is 2000 kDa. Brackets indicate fractions that were pooled for further immunoprecipitation analysis. Size population I is from WT cells and size population II is from R120G cells. D) Co-immunoprecipitation studies. a) Size population I from WT cells was immunoprecipitated with either anti-HspB1 (IPαB1) or anti-HspB5 antibody (IPαB5). Immunoprecipitated proteins-bound to proteinG-sepharose were washed in IPP150 buffer containing 150 mM NaCl before being processed for gel electrophoresis. After migration in SDS-PAGE, proteins were revealed in immunoblots probed with either anti-HspB1 or anti-HspB5 antibody. T: aliquot of cytosolic supernatant fractions before immunoprecipitation, IP: immunoprecipitated proteins, S: aliquot from supernatant after immunoprecipitation. b) Same as a) except that washes of the immunoprecipitated proteins were performed in IPP300 buffer containing 300 mM NaCl. c–d) same as a–b) but in this case analysis was performed with size population II from R120G cells. Autoradiographs of ECL-revealed immunoblots are presented.

We next analyzed the intracellular distribution of HspB1 and HspB5 in cell lysed in the presence of non-ionic detergent (Triton X-100). As seen in Fig. 2A, these proteins were mainly (97%) recovered in the 10,000×*g* supernatant fraction of WT cells, as was HspB1 in Neo cells. In contrast, a similar fraction of HspB5 and HspB1 (about 37% of total cellular content) was recovered in the pellet fraction of R120G cells. As control, the distribution of Hsp70 in the supernatant and pellet fractions was not significantly altered in the three cell types. Hence, even in a stable cell line, the R120G mutation was able to drive a fraction of the cellular content of HspB5 into insoluble aggregates. The fact that a similar fraction of HspB1 was also aggregated suggests a dominant effect of mutant HspB5 over HspB1. We also tested whether, in our cell system, endogenous HspB1 could modulate the level of mutant HspB5. WT and R120G cells were therefore transiently transfected with a DNA vector-based shRNA targeting HspB1 mRNA and its mismatch control. Immunoblot analysis presented in Fig. 2B, revealed that, in WT cells, the depletion of HspB1 had no significant effect towards the level of wild type HspB5. In contrast, in R120G cells, HspB1 almost complete withdrawal (93%) strongly decreased (down to 15%) HspB5 level. The phenomenon was partially abolished (back to 47%) in cells treated with the proteasome inhibitor MG132, hence confirming previous observations that HspB1 can chaperone mutant HspB5 and avoid its proteolytic degradation [64], [72].

Since HspB1 [58], [73] and HspB5 [60], [74] are oligomeric proteins, we analyzed their respective native size onto Sepharose 6B gel filtration columns. As previously described [58], in control Neo cells, HspB1 was recovered in three sub-populations (native sizes <150 kDa, 150–400 kDa and >400 kDa) (Fig. 2C). This particular oligomeric pattern was no more observed in WT cells since most of HspB1 (about 90%) shared an heterogenous native size, roughly comprised between 400 and 800 kDa, with HspB5 (Fig. 2C). In R120G cells, almost the same proportion of HspB1 shared the oligomerization profile of mutant HspB5 (see below for quantitative analysis). However, in this case the hetero-complex was of higher native size (up to about 900 kDa) compared to that formed with wild type HspB5 (Fig. 2C). This is probably linked to the fact that R120G HspB5 has been described to form larger oligomer than wild type HspB5 [41], [65]. To confirm the interaction of wild type and mutant HspB5 with HspB1, native immunoprecipitation studies (Fig. 2D) were performed using pooled fractions from the sizing columns (pools I in the case of WT cells and II in the case of R120G cells, see Fig. 2C). Anti-HspB1 (IPαB1) and anti-HspB5 (IPαB5) were used to immunoprecipitate the corresponding proteins (IP). The resulting immunoprecipitated proteins were then analyzed in immunoblots probed with either anti-HspB1 or anti-HspB5 antibody. In each case, HspB1 antibody quantitatively co-immunoprecipitated all HspB5 polypeptides present in the pooled fractions. Similar conclusions could be drawn when the antibody targeting HspB5 was used to perform IP. Aliquots of the total (T) and immunodepleted (S) pooled fractions were analyzed to verify if the immunoprecitation of the targeted protein was complete and to test the fate of the other protein partner. Since the immunodepleted supernatant fractions were devoid of the corresponding proteins, it was concluded that 100% of both HspB1 and HspB5 (wild type or mutant) present in the pooled fractions were interacting and formed chimeric complexes (Fig. 2Da and c). We then tested HspB1 and HspB5 interaction in presence of 300 mM NaCl. As seen in Fig. 2Db, interaction between HspB1 and HspB5 in the pooled fractions from WT cells was weakened by the 300 mM wash. Indeed, in the presence of high salt, a fraction (about 40%) of HspB5 or HspB1 corresponding partner was removed from the complex and recovered in the immunodepleted supernatant (S). The phenomenon was not observed when a similar analysis was performed using the pooled fractions from R120G cells (Fig. 2Dd). In this case the interaction was not altered by 300 mM NaCl, hence suggesting that it was tightened by the R120G mutation.

### Enhanced cellular resistance to oxidative stress induced by wild type HspB5 and oxidosensitivity mediated by R120G mutation

**Figure 3:**
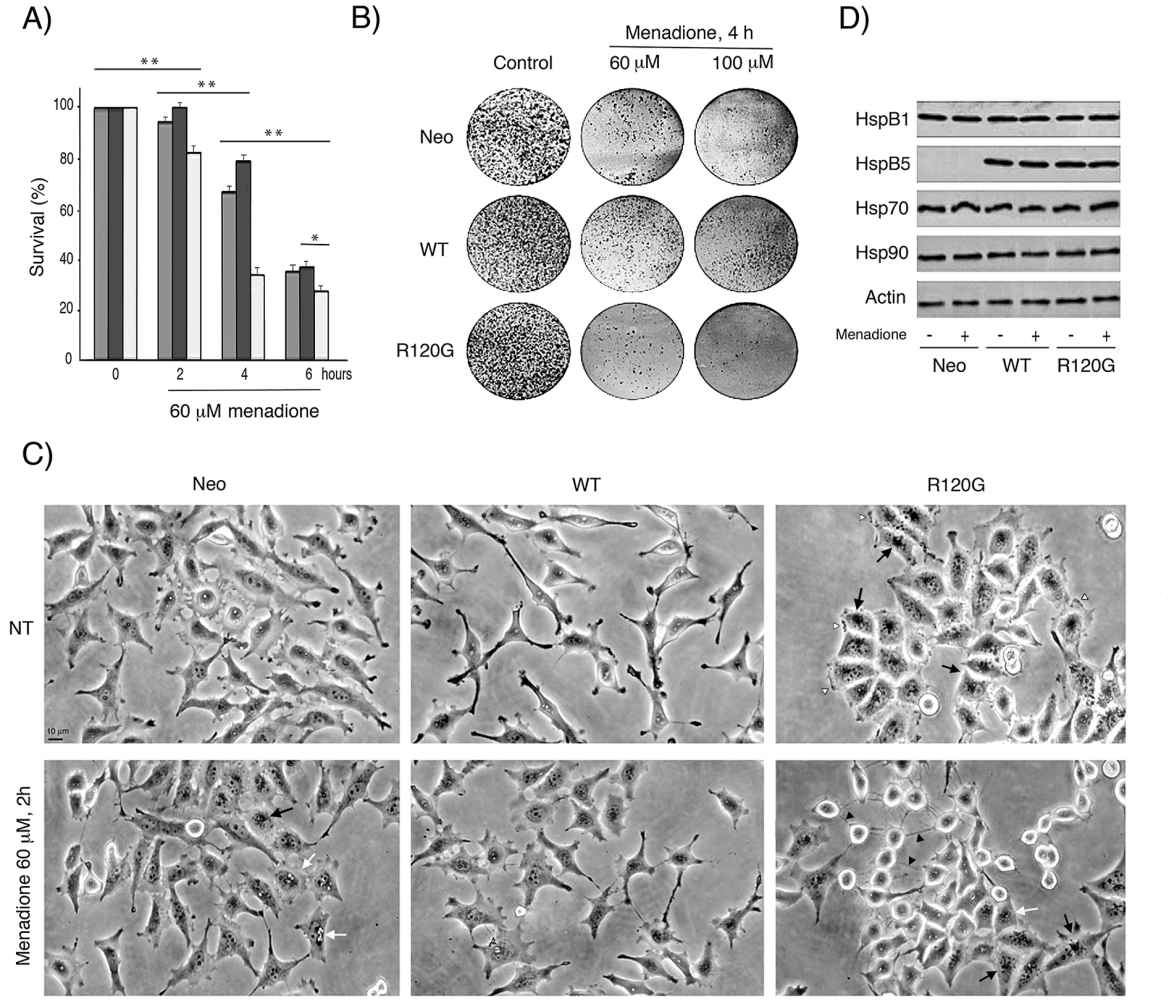
Enhanced oxidoresistance induced by wild type HspB5 and sensitivity mediated by the R120G mutation. Neo, WT or R120G cells were treated or not for different time periods with several concentrations of menadione. A) Crystal violet staining. The percentage of cell survival corresponded to the ratio of the relative absorbance of the different samples to that of untreated cells. Values are means ± SDM of three independent experiments. 2-way ANOVA indicates statistically significant differences in the survival to menadione treatment between Neo, WT and R120G cell lines, *P<0.05, **P<0.01. B) Clonogenic colony formation assay. The number of colonies was visually estimated. All experiments were performed in triplicate. C) Phase-contrast analysis of cell morphology. Before and after treatments, phase contrast analysis of the morphology of live cells was performed and photographs are presented. Bar: 10 µm. Black arrows: perinuclear granules; black arrowheads: filamentous bridges between cells; white arrowheads: membranous ruffles; white arrows: vacuoles. D) Immunoblot analysis of the level of HspB1, HspB5, Hsp70 and Hsp90 in menadione-treated Neo, WT and R120G cells.

We next analyzed the resistance of Neo, WT and R120G cells to oxidative conditions since this is a common stress encountered by cells expressing HspB5 (lens, muscle cells), as for example when they are exposed to UV light or chronic inflammation damages [75], [76]. Neo, WT and R120G cells were exposed for different time periods to 60 or 100 µM of menadione, a compound that generates intracellular reactive oxygen species (ROS) *via* redox-cycling [77]. Subsequently, their survival was determined using crystal violet staining, clonogenic colony formation assay and phase-contrast analysis of live cells (see Materials and Methods). It is seen in Fig. 3A,B, that WT cells were significantly more oxidoresistant than Neo cells while R120G cells displayed a pronounced sensitivity to menadione. Similar observations were made using Trypan blue staining of dead cells and after exposure to 100 µM of hydrogen peroxide (not shown). Morphological analysis, presented in Fig. 3C, revealed the accumulation of perinuclear vacuoles and granules in menadione-treated Neo cells that were not detected in WT cells. However, in spite of their apparent resistance to menadione, WT cells no more displayed an elongated morphology and had a more polygonal morphology; a phenomenon that could be related to the sensitivity of F-actin cytoskeleton to menadione induced oxidative stress [78]. About half of menadione-treated R120G cells had a dying morphology: they were detached from the substratum, had a rounded appearance and were linked to each other by filamentous bridges. The remaining living R120G cells were still attached and displayed a polygonal appearance. However, they had lost their dense membranous ruffles in the leading edges and were loaded with vacuoles and granules. Control experiments revealed no changes in the cellular content of HspB1 and HspB5 in response of menadione treatment as well as no stimulation of the level of two major ATP-dependent chaperones, Hsp70 and Hsp90 (Fig. 3D).

### The R120G mutation enhances HspB5 phosphorylation

**Figure 4:**
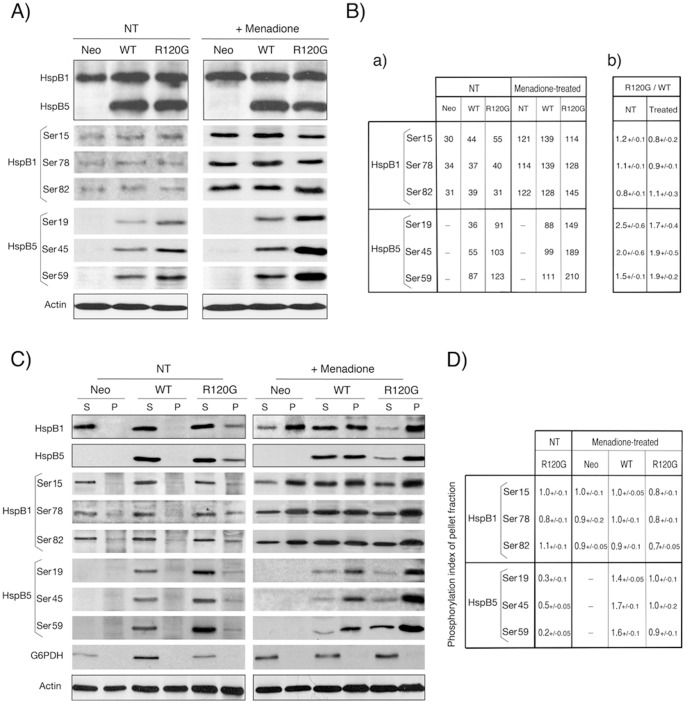
Analysis of HspB1 and HspB5 phosphorylation. A) Immunoblot analysis of total cellular proteins. HeLa cells were either kept untreated (NT) or treated for 2 h with 60 µM menadione. Total cellular protein extracts were analyzed in immunoblots probed with antibodies that are specific for HspB1 or HspB5 and for HspB1 phosphorylation at either serine 15 (Ser15), 78 (Ser78) or 82 (Ser 82) or HspB5 phosphorylation at either serine 19 (Ser19), 45 (Ser45) or 59 (Ser59) (see Materials and Methods). The corresponding levels of total HspB1, HspB5 and actin are shown as controls (marked by a dark line surrounding immunoblots). Autoradiographs of ECL-revealed immunoblots are presented. B) Quantitative analysis of HspB1 and HspB5 phosphorylation in untreated cells and following exposure to menadione. Ba) Level of HspB1 and HspB5 phosphorylation in the immunoblot presented in A. The level of actin was used as standard of equivalent protein loading. Bb) Modulation of the phosphorylation of HspB1 and HspB5 by the R120G mutation. The R120G/WT ratio of the different HspB1 and HspB5 phosphoserine sites was defined as the ratio between the level of phosphorylation in R120G cells to that observed in WT cells. The R120G/WT ratios were calculated from three independent experiments. NT: non-treated. Treated: menadione-treated. Standard deviations are indicated (n = 3). Note the positive effect of the mutation towards HspB5 phosphorylation. C) Immunoblot analysis of fractionated cells. Same as A) but in this case 10,000×*g* supernatant (S) and pellet (P) fractions were analyzed from cells lysed in TEM buffer containing 0.1% Triton X-100 and spun at 10,000 g for 10 min. As in A, the corresponding levels of total HspB1, HspB5 and actin are shown as controls (marked by a dark line surrounding immunoblots). Autoradiographs of ECL-revealed immunoblots are presented. D) Quantitative analysis. The phosphorylation index of pellet fraction was defined as the ratio of the percentage of the phosphorylated protein in pellet to the percentage of the protein in that particular fraction. A value of 1.0 indicates that phosphorylation is proportional to the level of the protein in the pellet fraction. A value>1.0 is indicative of an enhanced phosphorylation of the protein in the insoluble fraction. A value<1.0 is indicative of a decreased phosphorylation of the protein in the insoluble fraction. Standard deviations are indicated (n = 3). Note the decreased phosphorylation of mutant HspB5 in the pellet of untreated R120G cells and the stimulated phosphorylation of pelleted wild type HspB5 in response to oxidative stress.

HspB1 and HspB5 polypeptides contain three phosphorylated serine sites each [79]–[81] that are modulated by changes in the cellular environment and by stress [80] and which play major roles in their structural organization and function [58], [82]. We therefore tested HspB1 and HspB5 level of phosphorylation in Neo, WT and R120G cells exposed or not to 60 µM menadione for 2 h, a duration that corresponded to the maximal level of phosphorylation induced by this drug (not shown). It is seen in Fig. 4A and 4Ba,b that, in non-treated cells, expression of wild type or mutant HspB5 did not significantly change the level of phosphorylation of HspB1 as demonstrated by calculating the R120G/WT ratio, which is representative of the modulation of the phosphorylation of a define serine site in response to the mutation. In contrast to HspB1, the phosphoserine sites of HspB5 were stimulated by the R120G mutation (ratio of 1.5 to 2.5 depending on serine site, Fig. 4Bb). In response to menadione treatment, the phosphorylation of HspB1 serine sites was increased by about 4-fold. In the case of HspB5, the effect was less intense and showed a decreased intensity depending on the N-terminal position of the serine sites (Ser19: 2.4-fold, Ser45: 1.8-fold and Ser59: 1.3-fold). However, in oxidative conditions the R120G mutation still strongly enhanced HspB5 phosphorylation (R120G/WT ratio comprised between 1.7 and 1.9 depending of HspB5 serine site, Fig. 4 Bb). A similar observation was made when cells were exposed to hydrogen peroxide treatment (not shown).

Phosphorylation was further analyzed following cell fractionation in a 10,000×*g* supernatant and pellet (Fig. 4C). As expected, in non-treated Neo and WT cells, all the phosphorylated proteins were recovered in the soluble fraction. Analysis of the fraction of HspB1 and HspB5 present in the pellet fraction of R120G cells (see also figure 2A) was performed by comparing, in the immunoblots, the ratios between the signals given by the percentage of the phosphorylated protein in the pellet to that of the percentage of the protein in that particular fraction (defined as phosphorylation index of pellet fraction, Fig. 4D). In non-treated R120G cells, the fraction of HspB1 in the pellet fraction (37%, see also figure 2A) had a phosphorylation index close to 1.0 while that of mutant HspB5 was in the range, depending on the serine site, of 0.2 to 0.5. This indicates that mutant HspB5 in the pellet fraction is less phosphorylated than its soluble counterpart and that HspB1 level of phosphorylation is less affected by the redistribution of this protein in the pellet fraction. Analysis of menadione treated Neo cells first showed that a large fraction (71%) of the total cellular content of HspB1 was recovered in the pellet fraction. The phenomenon was less intense in WT cells (52%) and more drastic in R120G cells (86%). A similar observation was made for HspB5 in WT (50%) and R120G (79%) cells. Hence, the presence of these proteins in the pellet fraction correlated with the different sensitivity of these cells to oxidative stress (see figure 3). In response to menadione, HspB1 phosphorylation in Neo and WT cells was roughly proportional to the level of HspB1 in the fractions (index close to 1.0) while it was slightly decreased (0.7–0.8) in R120G cells. In contrast, HspB5 in WT cells showed a preferential phosphorylation in the pellet fraction (indexes from 1.4 to 1.7). The phenomenon was not observed in R120G cells since the high level of menadione-induced phosphorylation was close to be proportional (indexes from 0.9 to 1.0) to the level of HspB5 present in the soluble and pellet fraction (Fig. 4D).

### Analysis of HspB1-HspB5 phosphorylated oligomeric structures in normal and oxidative conditions

**Figure 5:**
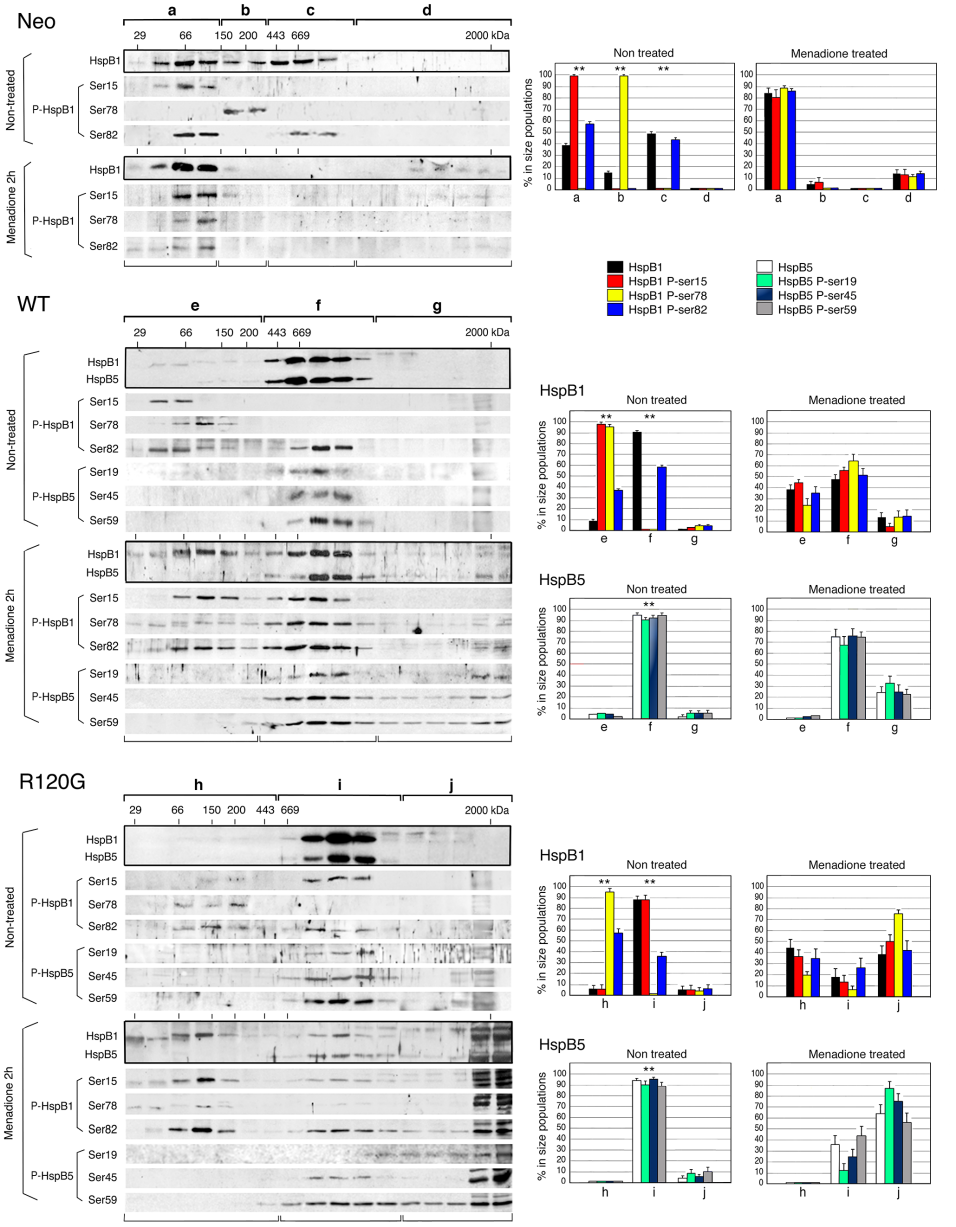
Analysis of the native size of phosphorylated HspB1 and HspB5. Non-treated as well as menadione-treated (60 µM, 2 h) Neo, WT and R120G cells were lysed and the S10,000×*g* supernatant fractions were applied to Sepharose CL-6B gel filtration columns as described in Materials and Methods. The presence of HspB1, HspB5 and their different phosphorylated isoforms (HspB1: Ser15, Ser78 and Ser82/HspB5: Ser19, Ser45 and Ser59) were detected in immunoblots of the collected fractions probed with the corresponding antibodies. The corresponding native size of total HspB1 or HspB5 is shown (marked by a dark line surrounding immunoblots). Autoradiographs of ECL-revealed immunoblots are presented. 29, 66, 150, 200, 443, 669 are gel filtration markers. Exclusion size of the column is 2000 kD. Quantitative analysis of the presence of HspB1 and HspB5 in different size domains of the column is presented (Neo cells: a, b, c and d; WT cells: e, f and g; R120G cells: h, i and j). Results are presented as percentage of HspB1, HspB5 and their different phosphorylated isoforms in the different size populations in regard to their amount in the S10,000×*g* supernatants. Standard deviations are indicated from three independent experiments. **P<0.01.

**Figure 6:**
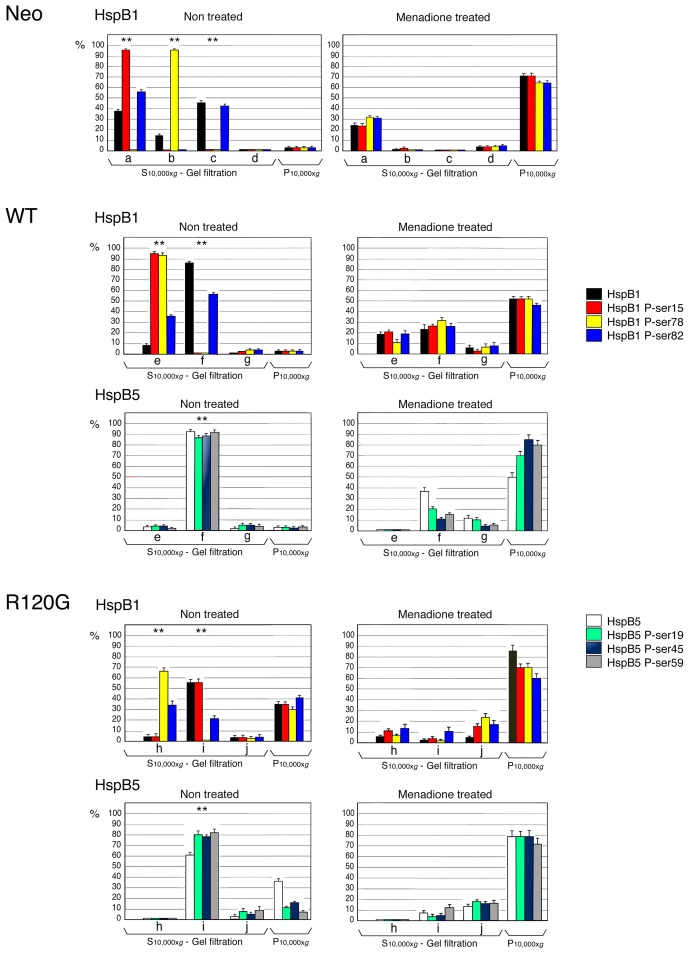
Quantitative analysis of the cellular distribution of phosphorylated HspB1 and HspB5. The data presented in Fig. 2A, 4 and 5 were used to calculate the percentage in regard to the total cellular content of HspB1, HspB5 and their different phosphorylated isoforms (HspB1: Ser15, Ser78 and Ser82/HspB5: Ser19, Ser45 and Ser59) in the different size populations (S10,000×*g*, gel filtration) and pellet fractions (P10,000×*g*). Size populations from gel filtration columns and color codes are the same as those described in Fig. 5. Standard deviations are indicated from three independent experiments. **P<0.01.

We next analyzed the phosphorylation of the different oligomeric structures of HspB1 and HspB5. Immunoblots from gel filtration analysis of the S10,000×*g* cytosolic fraction (see figures 2A and 4C) isolated from the Neo, WT and R120G cells were probed with antibodies that recognized HspB1 phosphoserines 15 (Ser15), 78 (Ser78) or 82 (Ser82) and HspB5 phosphoserines 19 (Ser19), 45 (Ser45) or 59 (Ser59) (Fig. 5). This figure also shows the percentage (with regard to their level in the S10,000×*g* cytosolic fraction) of these proteins in different size populations. Three size sub-populations of HspB1 have been defined in the S10,000×*g* cytosolic fraction of non-treated HeLa [58] or Neo cells which contains 97% of the total cellular contain of this protein. Each of them was characterized by a different set of phosphorylated serines (Fig. 5). Phosphoserine 15 was present only in the oligomers of less than 150 kDa together with 58% of the HspB1 soluble content of phosphoserine 82. Phosphoserine 78 was the only phosphoserine present in the medium sized oligomers (150–400 kDa). In contrast, the large oligomers contained the remaining soluble content of phosphoserine 82 (43%). In response to 2 h exposure to 60 µM menadione, the fraction of HspB1 and its phosphorylated forms (29 to 36%) that remained in the soluble fraction was mainly recovered as small oligomers (native size <150 kDa). Similar effects were induced by 100 µM of hydrogen peroxide (not shown). The percentage with regard to the total cellular content of HspB1 polypeptides, calculated in taking into account their presence in the 10,000×*g* pellet (see figure 4 C,D), is then presented in Fig. 6.

Similar analysis performed in WT cells (Fig. 5 and 6) revealed that the HspB1-HspB5 complex contained only one phosphorylated isoform of HspB1 (P-Ser82, 56% of the total cellular content of the modification). The very low level of soluble HspB1 (9%) that was not trapped in the complex and recovered in the small oligomers fraction was highly phosphorylated as it contained 36% of the total cellular content of phosphoserine 82 as well as most of phosphoserines 15 and 78 (95 and 93%, respectively, Fig. 6). Contrasting with these observations, the three phosphoserines of HspB5 were mostly recovered in HspB1-HspB5 complex. Upon exposure to menadione or hydrogen peroxide (not shown), a large fraction (48 to 80%) of HspB1 and HspB5 polypeptides was recovered in the pellet fraction (see figure 4C,D). Analysis of the remaining soluble HspB1-HspB5 complex revealed that it was partially dissociated. A large fraction of HspB1 (38% of soluble HspB1, Fig. 5) was back in the small oligomers range while a small fraction of HspB5 had the tendency to form large structures recovered in the void volume of the column. In this condition, the three phosphoserines of HspB1 were present in the remaining HspB1-HspB5 complex. P-Ser78 was abundant in this structure (65% of soluble form and 31% of total, see Fig. 5 and 6) together with 56% of soluble (28% of total) P-Ser15 and 51% of soluble (27% of total) P-Ser82. The remaining of these phosphorylated isoforms was recovered in the small oligomers and in large structures present in the void volume of the column and pellet fraction. The remaining HspB1-HspB5 complex still contained HspB5 phosphoisoforms. However, compared to HspB1, they were less abundant and mainly recovered in the pellet fraction (see figures 4, 5 and 6).

Analysis of non-treated R120G cells revealed the surprising presence of about 90% of cytosolic HspB1 P-Ser15 (56% of total cellular content, Fig. 6) in the complex formed between HspB1 and mutant HspB5. As mentioned above, this phosphorylation was clearly absent from the wild type HspB1-HspB5 complex (isolated from WT cells). HspB1 P-ser82 was still recovered (21% of total) in the mutant complex. As in WT cells, less than 10% of HspB1, not trapped in the complex, was in the form of highly phosphorylated small oligomers. However, they differed from those observed in WT cells since they were devoid of HspB1 phosphorylated at serine 15 (Fig. 5 and 6). As observed in WT cells, the three phosphoserines of HspB5 were mostly recovered in the complex. In that regard, it is interesting to note the weak phosphorylation of the relatively large fraction of mutant HspB5 (almost 40% of total) in the pellet fraction (Fig. 4C,D and 6). In response to menadione, HspB1-HspB5 complex was almost completely disrupted. A similar effect was induced by hydrogen peroxide (not shown). HspB1 was then recovered in the small oligomeric fractions and, together with HspB5, it had the tendency to form large structures or aggregates recovered in the void volume of the column and in the 10,000×*g* pellet fraction. This suggests that the mutant complex had lost its dynamic and controlled dissociation in stress conditions. Most of HspB1 phosphorylation was mostly recovered in small and large oligomers and pellet fraction while phosphorylated HspB5 was only detected in large oligomers and pellet fraction. Of interest, the remaining HspB1-HspB5 mutant complex still contained a small fraction of HspB1 P-ser15 and 82 and HspB5 P-ser45 and 59. However, in contrast to the remaining complex of menadione treated WT cells, it contained less of HspB1 P-ser78 and HspB5 P-ser19.

**Figure 7:**
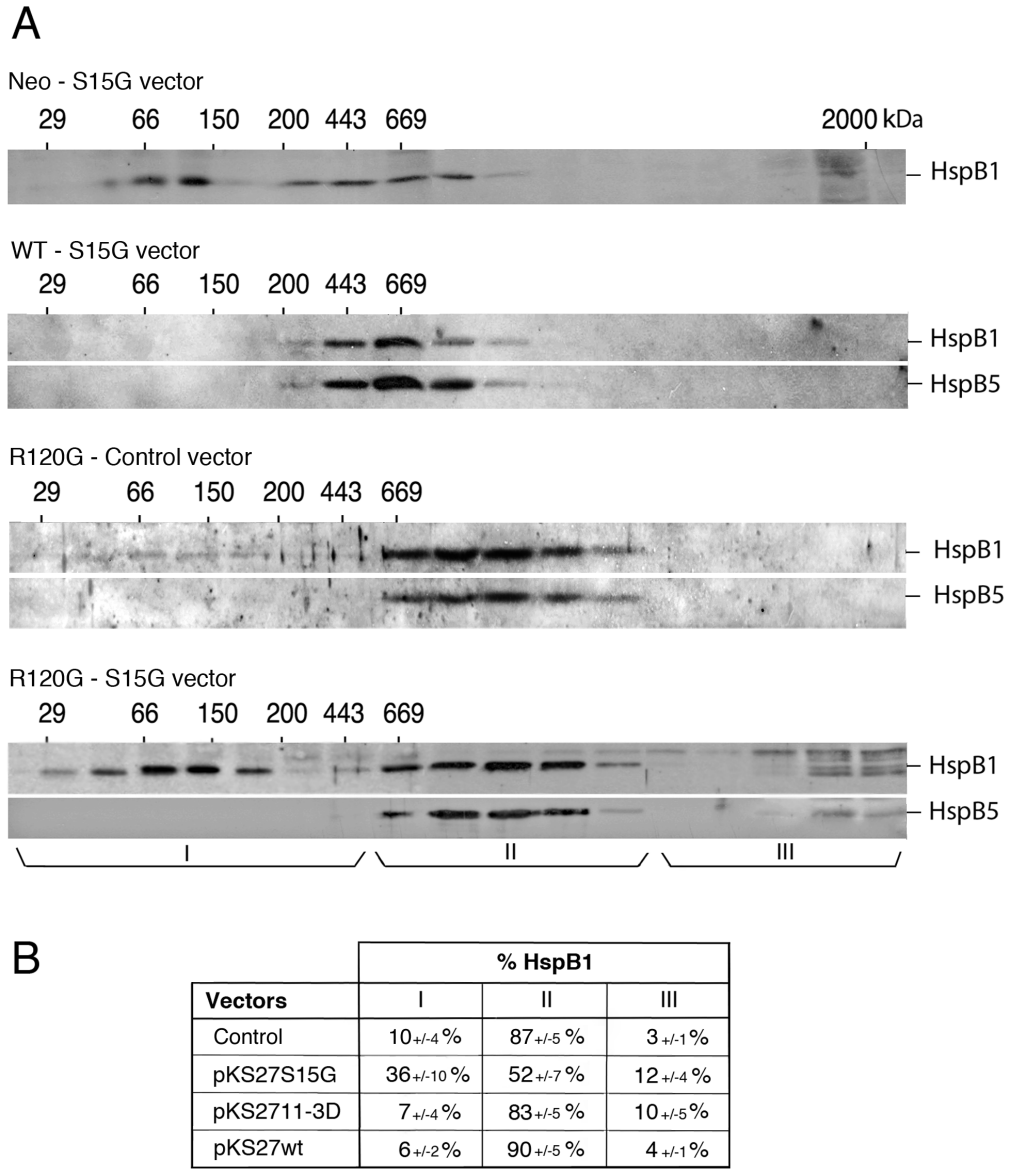
Effect of HspB1 serine 15 phosphorylation on HspB1-HspB5 native size. A) Neo, WT and R120G cells were transiently transfected with pKS27S15G vector (phosphoserine 15 of HspB1 replaced by glycine, S15G vector; see Materials and methods) and HspB1 as well as HspB1/HspB5 native sizes were subsequently analyzed as described in Fig. 5. Results obtained from R120G cells transiently transfected with the corresponding empty vector are also presented (control vector). Three size domains (I, II, and III) were defined in the R120G column fractions that corresponded to the h, i and j fractions indicated in Fig. 5 and 6. Autoradiographs of ECL-revealed immunoblots are presented. 29, 66, 150, 200, 443, 669 are gel filtration markers. Exclusion size of the column is 2000 kD. B) Quantitative analysis. The distribution of HspB1 between size domains I, II and III of transiently transfected R120G cells is shown and expressed as percentage of the HspB1 content in the 10,000×*g* supernatant loaded on the column. The following vectors were used: pKS control, pKS27S15G (phosphoserine 15 of HspB1 replaced by glycine), pKS2711-3D (the three phosphoserine sites of HspB1 replaced by aspartic acid) and pKS27wt (wild type HspB1). Standard deviations are indicated from three independent experiments.

Since HspB1 phosphorylation at serine 15 is the major modification observed between wild type and mutant HspB1-HspB5 complexes, we tested how critical this phosphorylation could be in relation to HspB1-HspB5 mutant complex. We previously showed that transfected exogenous mutant of HspB1 polypeptide can interact with endogenous HspB1 and modulate its structural organization [58]. Hence, Neo, WT and R120G cells were transiently transfected with a DNA vector encoding HspB1 non-phosphorylatable mutant where serine 15 was replaced by a glycine residue (S15G mutant) (Fig. 7). Following transfection of Neo cells, the overall oligomerization profile of HspB1 (exogenous plus endogenous) was not drastically changed. This may be due to the fact that only the small oligomers of endogenous HspB1 are phosphorylated at the level of serine 15. Similarly, the S15G mutant had no significant effect towards the level of HspB1-HspB5 complex from transiently transfected WT cells. In contrast, following transfection of R120G cells, a fraction of HspB1 (36%) shifted from the mutant complex towards small oligomeric fractions suggesting that the transiently expressed mutant had destabilized HspB1-mutant HspB5 interaction (Fig. 7A). Transient transfection of HeLa cells with a vector encoding HspB1 phospho-mimicry mutant was without noticeable effects in WT or R120G cells (Fig. 7B). Serine 15 phosphorylation may therefore strengthen HspB1 interaction with mutant HspB5 but does not appear essential for the structural organization of wild type HspB1-HspB5 complex.

## Discussion

To study the interaction between HspB1 and HspB5 in a defined cellular environment, HeLa cells were used since they constitutively express a high level of HspB1 but do not, or only weakly, express other interacting sHsps, particularly HspB5 and HspB6, which are known to form hetero-oligomeric complexes with HspB1 [63], [70], [71]. Genetically modified cells that stably express similar levels of either wild type or R120G mutated HspB5 were obtained. Of interest, HspB5 level of expression was close to that of endogenous HspB1. Another interesting characteristic was that more than 60% of HspB5 mutant expressed in these cells was recovered in a soluble form. This rather low level of aggregation suggests that the selected clones are probably adapted to the presence of the mutant protein. Indeed, R120G HspB5 polypeptide is well known for its drastic aggregation prone property [39], [41], [83]; a phenomenon particularly intense in cells devoid of HspB1 expression [65], [84]. We have taken advantage of this cell system to analyze the consequences of the expression of the myopathy- and cataract-causing R120G HspB5 mutant in cells that already express HspB1. It also represents a cellular model that mimics the early phases of the pathological state before aggresomes of R120G mutated HspB5 accumulate. Expression of wild type HspB5 often correlated with an elongated cellular morphology while expression of the R120G mutant induced the accumulation of membranous ruffles in the leading edges. These effects, which are probably linked to cytoskeleton reorganization, did not change the level of expression of stress markers, such as Hsps. R120G mutant expressing cells were also characterized by perinuclear granules, a phenomenon concomitant with the presence of almost 40% of the cellular content of both HspB1 and HspB5 in the particulate fraction upon cell lysis. Another dominant effect of HspB5 was observed at the level of the native size of HspB1. In presence of type HspB5, HspB1 oligomerization profile was lost and almost 90% of the total cellular amount of this protein interacted with HspB5. HspB5 R120G mutant acted similarly and interacted with HspB1 in a complex of slightly higher molecular mass. Analysis of the resistance to salt-mediated dissociation revealed that HspB1-HspB5 interaction was strengthened by the R120G mutation. This suggests that the interaction was more rigid and less dynamic. The other side of the coin concerned the protective effect of HspB1 towards HspB5. Previous reports mentioned that HspB1 could stabilize mutant HspB5 [64], [65], [72] and attenuate the formation of inclusion bodies induced by expression of R120G HspB5 [85]. Using an RNAi approach, we confirmed that, in our cell system, HspB1 attenuates its ubiquitin-proteasome dependent clearance.

We report here that cells expressing wild type HspB5 were more oxidoresistant than control cells while those expressing mutant HspB5 displayed a drastically enhanced sensitivity to oxidative stress. HspB5 molecular mechanism that drives oxidoresistance is not yet known. It could result of cytoskeletal protection, since this is a major cellular target rapidly altered in cells exposed to menadione [78], or modulation of intracellular redox status since HspB1 and HspB5 have been shown to decrease ROS levels [12], [13] and up-regulate the activity of different anti-oxidant enzymes, particularly G6PDH [14]. Moreover, an effect towards HspB1, which gains in chaperone activity once it interacts with HspB5 [59], cannot be excluded (see below). On the other hand, the enhanced sensitivity mediated by the R120G mutation could be a direct consequence of the altered HspB1-HspB5 complex that cannot dissociate in a dynamic and controlled way and the lack of chaperone activity of mutant HspB5 [42]. This may lead to a defective recognition of oxidized polypeptides by these chaperones.

Analysis of the phosphorylation status pointed to the increased phosphorylation of HspB1 and HspB5 in response to menadione treatment. This study also confirmed the already described hyperphosphorylation of mutant HspB5 [83], [86] in spite of the fact that this polypeptide is in a chimeric complex with HspB1. In that regard, it is interesting to note the R120G mediated enhanced phosphorylation was more intense for the serine sites that are close to the N-terminus; this could disturb hexamer contacts and favor HspB5 oligomer heterogeneity [87]. Two-dimensional immunoblots analysis confirmed the acidic property of the isoforms suggesting that most R120G HspB5 molecules could be phosphorylated (data not presented here). Further analysis following fractionation of the different cell types revealed that the presence of these proteins in the pellet fraction correlated with their sensitivity to oxidative stress. Analysis of the phosphoserine sites of either HspB1 or HspB5 enlightened the different and complex patterns of phosphorylation of these polypeptides, particularly after oxidative stress, which drove a large fraction of these proteins in the particulate fraction. For example, it was first observed that, in the different untreated cells as well as in menadione-treated Neo and WT cells, HspB1 phosphorylation was roughly proportional to its level in the fractions. However, phosphorylation was less intense in the pellet fraction of menadione-treated R120G cells. In menadione-treated WT cells, but not in R120G cells, a preferential phosphorylation of HspB5 in the pellet fraction was observed. Moreover, we noticed that mutant HspB5 in the pellet fraction of untreated R120G cells was less phosphorylated than its soluble counterpart.

Further analysis of the phosphorylation of the different oligomeric structures of HspB1 and HspB5 confirmed the complex nature of these modifications. As already described, in HeLa as well as in control neo cells HspB1 is characterized by three sub-populations based on their serine sites specific phosphorylation and native size [58]. However, in WT cells this structural organization was no more observed since most HspB1 interacted with HspB5 resulting in the formation of a large oligomeric complex which surprisingly contained only one HspB1 phosphorylated isoform (phosphoserine 82). Hence, in spite of the fact that the N-terminal domain of HspB1 consisting of amino acids 1–124, which bears the phosphoserine sites, has been reported to not interact with HspB5 [88], our observations suggest that its recognition by MAPKAPK2,3 kinase is potentially impaired. Contrasting with these observations, the partial dissociation of HspB1-HspB5 complex in response to menadione was associated with the presence of the three HspB1 phosphoisoforms in the remaining complex, hence suggesting a profound reorganization of this chimeric complex. In both normal and oxidative conditions, HspB5 phosphoisoforms were mainly localized in the HspB1-HspB5 complex. This suggests that HspB1 does not interfere with the kinase accessibility of HspB5 N-terminal domain. Of interest, in untreated WT cells, the small fraction (less than 10%) of HspB1 that was not interacting with HspB5 was recovered in small oligomers that differed in their phosphorylation from those observed in control Neo cells devoid of HspB5 expression. Consequently, the formation of HspB1-HspB5 complex indirectly generated the formation of a new-type of highly phosphorylated small HspB1 homo-oligomers that could play a role in the enhanced oxidoresistance of WT cells through their ability to interact with G6PDH [89].

The R120G mutant altered HspB1-HspB5 structural organization in such a way that most of the cellular content of HspB1 phosphoserine 15 was now recovered in the complex while this modification was not present in the complex formed with wild type HspB5. In contrast, no major changes in HspB1 phosphoserines 78 and 82 distribution were noticed. In spite of the fact that HspB1-mutant HspB5 complex was almost completely disrupted by oxidative stress, it is interesting to note that, as in WT cells, the remaining complex contained the three phosphoisoforms of HspB1. This suggests that in oxidative conditions, the N-terminal part of HspB1 can be freely recognized by the corresponding kinase. Moreover, the partial disruption of the mutant complex in cells transiently expressing HspB1 serine 15 non-phosphorylatable mutant suggests that serine 15 phosphorylation could stabilize HspB1 interaction with mutant HspB5. It is not known whether the absence of serine 15 phosphorylation in the wild type complex is due to the masking of this serine site or reflects its uselessness nature for stabilization.

Within the limitation that this study has been made in a single set of HeLa-derived cell lines, the observations reported here illustrate the complex nature of the interaction between HspB1 and HspB5. It is also not known if similar interacting behavior occurs in other types of cells. Moreover, we cannot exclude that the low level of HspB6 expression detected in HeLa cells may modulate, at least to a certain extent, the structural organization of the complexes formed by HspB1 and HspB5. Further studies will have to clarify these points. Hence, the major hallmarks induced by the R120G mutation at the level of the HspB1-HspB5 complex in HeLa cells are: larger native size, enhanced dissociation and aggregation in oxidative condition as a probable consequence of the enhanced rigidity of the mutant complex, and phosphorylation of HspB1 serine 15 in the complex to stabilize HspB1 interaction with mutant HspB5.

## Materials and Methods

### Vectors, cells and reagents

pIRESneo vector bearing human HspB5 coding sequence (pIREShαBcry) has already been described [66]. Stratagen QuickChange™ Site-Directed Mutagenesis Kit (Agilent Technologies, Massy, France) was used to create the R120G mutation in HspB5 coding sequence and to generate pIREShmutαBcry-R120G vector. PCR mutagenic primers were: a) antisense 5′-GGGATCCGGTATTT**CCC**GTGGAACTCCCTGG-3′ and b) sense 5′-CCAGGGAGTTCCAC**GGG**AAATACCGGATCCC-3′ (mutant codons are indicated in bold). HeLa cells were stably transfected with wild type and mutant vectors as well as with pIRESneo plain vector. Several G418 clones were obtained that expressed different levels of either wild type or mutant HspB5. Two clones were selected that expressed identical levels of wild type (designated WT) and mutant HspB5 (designated R120G). Controls cells were indicated as Neo. Parental, Neo, WT and R120G cells were grown at 37°C in the presence of 5% CO_2_ in Dulbecco's modified Eagle medium containing 10% fetal calf serum (Invitrogen, Abingdon, UK) and supplemented with 500 µg/ml of G418 (Sigma, St-Louis, MO). DNA vector-based shRNA targeting HspB1 mRNA as well as the mismatch control have already been described [67]. Transient transfection assays were performed using Invitrogen lipofectamine reagent (Invitrogen, Abingdon, UK), using pKS27wt (wild type HspB1), pKS27S15G (HspB1 phosphoserine 15 non-phosphorylatable mutant: serine 15 replaced by glycine) and pKS2711-3D (HspB1 phosphomimicry mutant: serines 15, 78 and 82 replaced by aspartic acid) DNA vectors as already described [58], [67]. Anti-human Hsp70, HspB6 (Hsp20), HspB1 and HspB5 antibodies as well as those that specifically recognize phosphorylated HspB1 (at either serine 15, 78 or 82) or phosphorylated HspB5 (serine 19, 45 or 59) were from Enzo-Covalab (Villeurbanne, France). Alexa Fluor™ 488 Phalloidin was from Molecular Probes/Interchim (Montluçon, France) and anti-vimentin antibody was from Dako (Glostrup, Denmark). Hoechst 33258, MG132, menadione and hydrogen peroxide were from Sigma (St Louis, MO). FITC-conjugated goat-anti mouse and TRITC-conjugated goat anti-rabbit secondary antibodies were Santa Cruz Biotechnologies-Clinisciences (Montrouge, France). Recombinant HspB1 and HspB5 were from Enzo-Covalab (Villeurbanne, France).

### Immunoblotting

One and two-dimensional immunoblots were performed as already described [43], [68]. They were probed with antibodies specific to the targeted proteins before being incubated with either goat anti-mouse or anti-rabbit immunoglobulin conjugated to horseradish peroxidase (Santa Cruz Biotechnology-Tebu, Le Perray en Yvelines, France) and subsequently revealed with ECL (Amersham Corp., Buckinghamshire, UK). Autoradiographs were recorded onto X-Omat LS films (Eastman Kodak Co, Rochester, USA). Films were scanned using a 4990 Epson film scanner and analyzed for quantification with ImageJ software™ (NIH, Bethesda). The duration of the exposure was calculated as to be in the linear response of the film.

### Immunofluorescence analysis

HeLa cells (10^4^/cm^2^) growing on glass cover slips were fixed for 10 min with freshly prepared 3.7% formaldehyde pH 7.0 in Phosphate buffered saline (PBS), before being permeabilized for 5 min in ice-cold acetone. F-actin was stained for 20 min with Alexa Fluor™ 488 Phalloidin (5U per ml of PBS) and vimentin was detected by incubating the coverslips for one hour with monoclonal anti-vimentin antibody (diluted 1/200 in PBS containing 0.1% bovine serum albumin IgG free). Other cover-slips were used to detect HspB1 and HspB5 using the corresponding anti-HspB1 monoclonal and anti-HspB5 polyclonal antibodies (diluted 1/100). After washing, HspB1, HspB5 and vimentin staining were revealed by incubating the cover-slips for one hour with FITC- and TRITC-conjugated goat-anti mouse or rabbit immunoglobulins (1/200 in TBS-Tween containing 0.1% IgG-free bovine serum albumin). Control experiments performed with non-immune sera or only the second antiserum confirmed that all detectable HspB1, HspB5 and vimentin fluorescences were specific. Hoechst 33258 staining was used to detect nuclei. The stained cells were then examined and photographed using in an LSM510 laser scanning confocal microscope (Carl Zeiss AG, Oberkochen, Germany) using a 63$ (numerical aperture, 1.4) Zeiss Plan Neo Fluorobjective. Illumination sources were 488 nm and 543 nm. To avoid cross talk between the different fluorochromes, the multitrack recording module was used, which allows a sequential acquisition of each channel. Individual as well as merge analysis were performed. Overlap and Pearson's coefficients were determined using the JACoP plug-in [69] of ImageJ (NIH, Bethesda, USA). Computerized image analysis was performed using AxioVison LE software (Carl Zeiss MicroImaging GmbH, Germany).

### Cell viability assays

a) Cristal violet staining. Neo, WT and R120G cells as well as cells that were transiently transfected for 48 h were seeded in 96-wells plates (7.5×10^3^/well) and treated 12 h later for different time periods with several concentrations of menadione (Sigma, St Louis, MO) before being analyzed as previously described [27]. The percentage of cell survival corresponded to the ratio of the relative absorbance of the different samples to that of untreated cells. b) Clonogenic colony formation assay. Briefly, 24 h after being exposed or not to menadione, cells were incubated at 37°C for ten additional days. They were rinsed in PBS, and stained with 2% methylene blue in 50% ethanol. The number of colonies was then visually estimated. c) Trypan blue staining assay. 24 h before the experiment, about 5.10^5^ cells were distributed in 6-well dishes before being treated with menadione and subsequently counted using a Malassez counting chamber. All experiments were done in triplicate. d) Phase contrast analysis of the morphology of live cells was performed using a Nikon TMS inverted microscope. Images were recorded with a Nikon D300 digital camera coupled to a Nikon PFX device. RAW images were developed using Nikon Capture NX2 software (Nikon France, Champigny sur Marne, France).

### Cell fractionation and gel filtration analysis

HeLa cells were washed in ice-cold PBS pH 7.4, scrapped from the dish and pelleted 5 min at 1000×*g*. They were then either directly resuspended in SDS sample buffer and boiled or resuspended in a cold TEM lysis buffer made of 20 mM Tris-HCl pH 7.4; 20 mM NaCl; 5 mM MgCl_2_; 0.1 mM EDTA and 0.1% Triton X100. The lysates were centrifuged at 10,000×*g* for 10 min. SDS sample buffer (1× or 5×) was added to the resulting pellets and supernatants to obtain samples that had similar volumes. Samples were then processed for SDS-PAGE and immunoblot analysis using appropriate antibodies. Gel filtration analysis was as already described [58], [67]. Briefly, 2.10^7^ HeLa cells were lysed as above using Triton X100. The 10,000×*g* supernatant was applied onto a sepharose 6B gel filtration column (1×100 cm) (Pharmacia, Ullis, France) equilibrated and developed in TEM lysis buffer devoid of Triton X100 and calibrated with a molecular weight markers kit (Kit for Molecular Weights 29,000–700,000 for Gel Filtration Chromatography, Sigma, St Louis, MO). The fractions eluted from the column were pooled two by two and analyzed by immunoblotting.

### Co-Immunoprecipitation experiments (Co-IP)

2 ml samples of the pooled column fractions were incubated (4 h at 4°C) with either HspB1 antibody (goat polyclonal anti-Hsp27 (HspB1) antibody, Santa Cruz Biotechnologies-Clinisciences, Montrouge, France) or anti-HspB5 (mouse monoclonal, Enzo-Covalab, Villeurbanne, France) and then for 4 h at 4°C with protein G sepharose (GE Healthcare, Vélizy, France; 50 µl per sample of a 50% bead slurry in IPP150 buffer: 20 mM Tris-HCl pH 8; 150 mM NaCl; 0,05% NP-40). Samples were briefly centrifuged (5,000×*g*, 30 s) and after several washings of sepharose beads with either IPP 150 buffer or IPP300 buffer (same as IPP150 but containing 0.3 M NaCl), immunoprecipitated proteins were eluted with boiling SDS-sample buffer. Detection of co-immunoprecipitated proteins was performed in immunoblots probed with the corresponding antibodies. Percentage of the proteins present in the immunoprecipitated complexes was estimated by comparing, in immunoblots, the level of the targeted proteins present in the pooled column fractions before and after the immnoprecipitation step.

### Statistical analysis

Cell morphology. Due to the elongated morphology that characterizes WT cells, the biggest dimension of Neo, WT and R120G cells was measured (in the pictures) using ImageJ software (NIH, Bethesda, USA) and compared. Mean, Standard Deviation of the Mean (SEM) and Standard Deviation (SD) of the biggest dimension of cells was determined using Prism 6.0b Software (GraphPad, La Jolla, USA). Statistical analysis of cell number was performed using a one-way ANOVA test within a time point analysis. A 2-way ANOVA test within and between time points was performed to compare the survival to menadione treatment of Neo, WT cells and R120G cell lines (Prism Software, GraphPad, La Jolla, USA). Immunoblots were quantified using ImageJ software (NIH, Bethesda, USA) and Mean and SD were determined using Prism 6.0b Software (GraphPad, La Jolla, USA).

## Supporting Information

Data S1 **Immunofluorescence analysis.** Neo, WT and R120G cells were processed for detection of F-actin and intermediate filament protein vimentin as described in Materials and Methods. Bar: 10 µm. In R120G cells, the arrowhead and arrow point to the F-actin spherical and collapsed intermediate filament networks, respectively.
(TIF)

Data S2 **Aggregated appearance of HspB5 R120G mutant in transiently transfected HeLa cells.** Parental HeLa cells were transiently transfected with pIRESneo vector encoding either wild type (pIREShαBcry)(A) or R120G mutant (pIREShmutαBcry-R120G)(B) HspB5. 48 h after transfection cells were processed for immunofluorescence analysis using anti-HspB5 antibody as described in Experimental procedures. Bar: 10 µm.
(TIF)
