# Supplementary figures and images for: Analysis of the Dominant Effects Mediated by Wild Type or R120G Mutant of αB-crystallin (HspB5) towards Hsp27 (HspB1)
Source: PLoS One. 2013 Aug 12;8(8):e70545. doi: 10.1371/journal.pone.0070545 (PMC3741289; doi:10.1371/journal.pone.0070545)

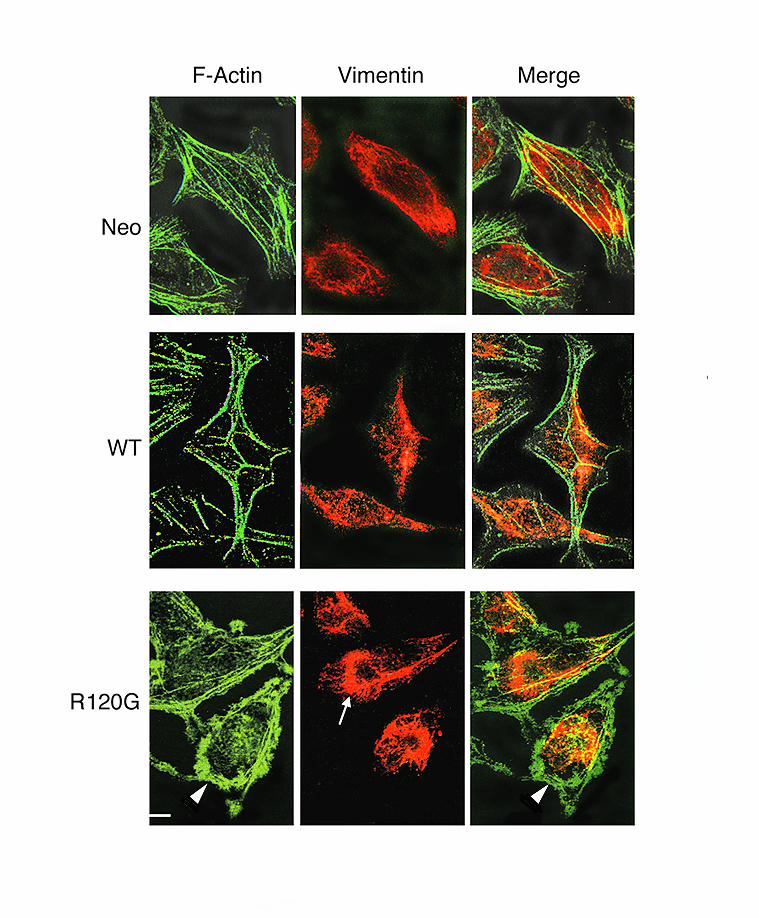

Supplement: Data S1 — Immunofluorescence analysis. Neo, WT and R120G cells were processed for detection of F-actin and intermediate filament protein vimentin as described in Materials and Methods. Bar: 10 µm. In R120G cells, the arrowhead and arrow point to the F-actin spherical and collapsed intermediate filament networks, respectively. (TIF) [file pone.0070545.s001.tif]

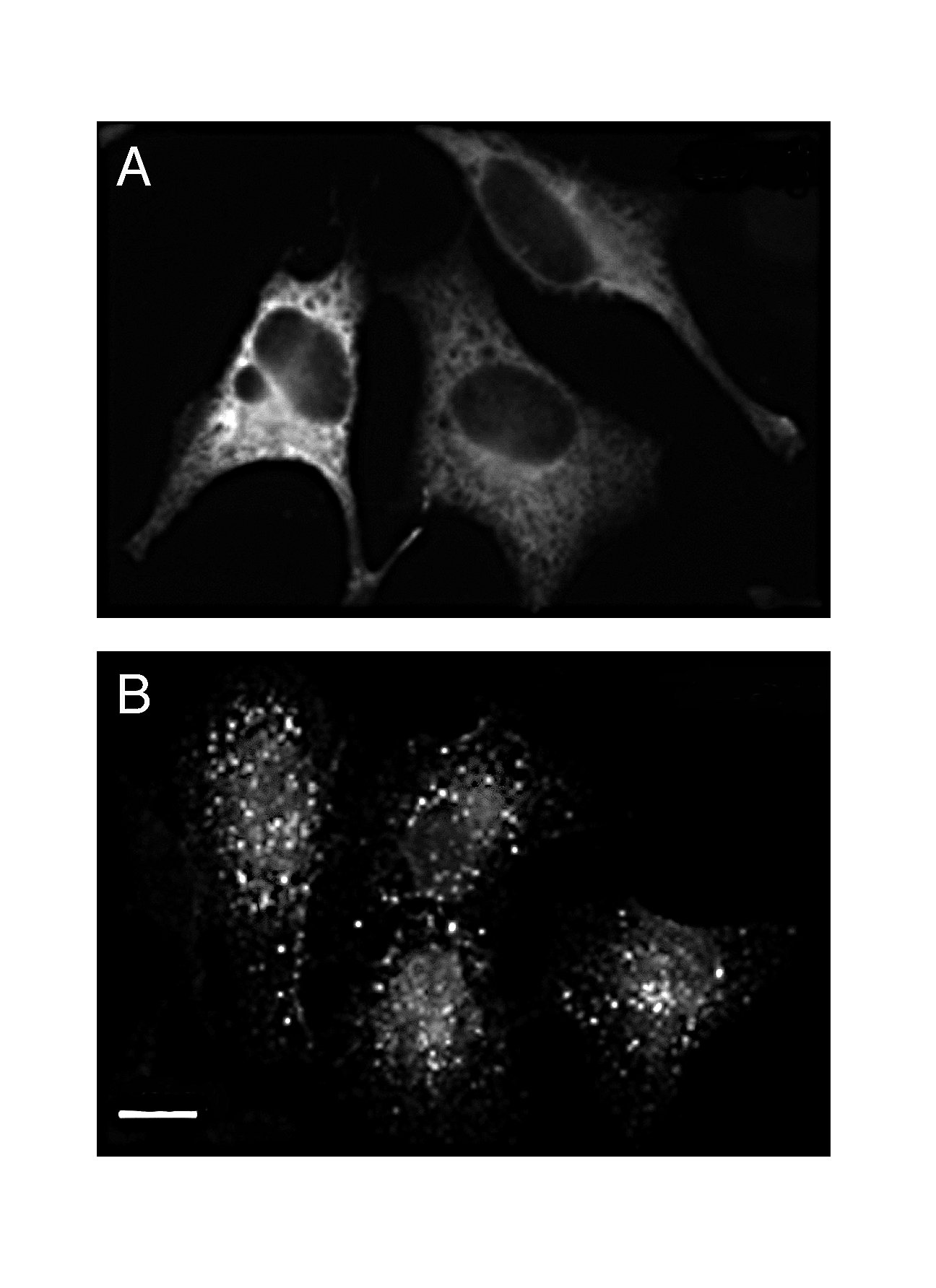

Supplement: Data S2 — Aggregated appearance of HspB5 R120G mutant in transiently transfected HeLa cells. Parental HeLa cells were transiently transfected with pIRESneo vector encoding either wild type (pIREShαBcry)(A) or R120G mutant (pIREShmutαBcry-R120G)(B) HspB5. 48 h after transfection cells were processed for immunofluorescence analysis using anti-HspB5 antibody as described in Experimental procedures. Bar: 10 µm. (TIF) [file pone.0070545.s002.tif]
